# MicroRNA-451a overexpression induces accelerated neuronal differentiation of Ntera2/D1 cells and ablation affects neurogenesis in microRNA-451a^-/-^ mice

**DOI:** 10.1371/journal.pone.0207575

**Published:** 2018-11-21

**Authors:** Christa Trattnig, Muammer Üçal, Carmen Tam-Amersdorfer, Angela Bucko, Ulrike Zefferer, Gerda Grünbacher, Markus Absenger-Novak, Kristin Anna Öhlinger, Klaus Kraitsy, Daniel Hamberger, Ute Schaefer, Silke Patz

**Affiliations:** 1 Research Unit for Experimental Neurotraumatology, Department of Neurosurgery, Medical University, Graz, Austria; 2 Institute for Pathophysiology and Immunology, Medical University, Graz, Austria; 3 Core Facility Microscopy, Centre of Medical Research, Graz, Austria; University of Colorado Boulder, UNITED STATES

## Abstract

MiR-451a is best known for its role in erythropoiesis and for its tumour suppressor features. Here we show a role for miR-451a in neuronal differentiation through analysis of endogenous and ectopically expressed or silenced miR-451a in Ntera2/D1 cells during neuronal differentiation. Furthermore, we compared neuronal differentiation in the dentate gyrus of hippocampus of miR-451a^-/-^ and wild type mice. MiR-451a overexpression in lentiviral transduced Ntera2/D1 cells was associated with a significant shifting of mRNA expression of the developmental markers Nestin, βIII Tubulin, NF200, DCX and MAP2 to earlier developmental time points, compared to control vector transduced cells. In line with this, accelerated neuronal network formation in AB.G.miR-451a transduced cells, as well as an increase in neurite outgrowth both in number and length was observed. MiR-451a targets genes MIF, AKT1, CAB39, YWHAZ, RAB14, TSC1, OSR1, POU3F2, TNS4, PSMB8, CXCL16, CDKN2D and IL6R were, moreover, either constantly downregulated or exhibited shifted expression profiles in AB.G.miR-451a transduced cells. Lentiviral knockdown of endogenous miR-451a expression in Ntera2/D1 cells resulted in decelerated differentiation. Endogenous miR-451a expression was upregulated during development in the hippocampus of wildtype mice. In situ hybridization revealed intensively stained single cells in the subgranular zone and the hilus of the dentate gyrus of wild type mice, while genetic ablation of miR-451a was observed to promote an imbalance between proliferation and neuronal differentiation in neurogenic brain regions, suggested by Ki67 and DCX staining. Taken together, these results provide strong support for a role of miR-451a in neuronal maturation processes *in vitro* and *in vivo*.

## Introduction

MiR-451a is a distinct dicer-independent miRNA that has been extensively studied in brain malignancies such as glioblastoma multiforme. MiR-451a expression has been shown to be downregulated in glioblastoma cell lines. Forced overexpression of miR-451a in these cells, moreover, results in decreased cell proliferation and viability, consistent with its tumour suppressor role [1]. Cancer stem cells, however, lose their stemness characteristics upon miR-451a overexpression [2, 3]. Gal et al have shown that miR-451a specifically causes CD133^+^ cancer stem cells to differentiate into CD133^-^ cells [4]

A regulatory role of miR-451a in brain has also been suggested by our own previous studies showing that microparticles derived from cerebrospinal fluid (CSF) of patients during the acute stages of traumatic brain injury (TBI) are specifically enriched in miR-451a [5]. These observations are consistent with a study showing rapid down-regulation of miR-451a in a rat model of traumatic brain injury [6], which could potentially be evoked by the release of miR-451a enriched microparticles into the cerebrospinal fluid [5]. Application of microparticles isolated from the CSF of TBI patients to Ntera2/D1 (NT2) cell cultures, led to a downregulation of CD133 and FGFR1 mRNA expression, which could be blocked by miR-451a antisense oligonucleotides, indicating miR-451a to be the main effector [5]. CD133 and FGFR1 have both been associated with cell proliferation [7–9] and miR-451a-mediated down-regulation of them in Ntera2/D1 (NT2), a cell line that differentiates along the neuroectodermal lineages after exposure to retinoic acid (RA) [10], might indicate a role of miR-451a in the onset of early differentiation of this neurogenic cell line.

A role of miR-451a in the regulation of stem cell differentiation has previously been established in erythropoiesis. MiR-451a was shown to drive hematopoietic stem cell differentiation into the erythroid lineage in the absence of extrinsic factors [11] leading to erythrocyte maturation [12, 13].

Based on these general findings, we hypothesized that miR-451a might regulate the early onset of neuronal differentiation. We tested this hypothesis *in vitro* by overexpression of miR-451a in Ntera2/D1 cells and by analysing the effect of the miRNA on retinoic acid induced neuronal differentiation of this cell line.

Our results indicate that miR-451a drives the maturation of neural stem cells. Retinoic acid (RA)-induced differentiation of NT2 cell-derived neurospheres was significantly accelerated by miR-451a overexpression. This was substantiated by earlier upregulation of various neurogenic markers, as well as by morphological analyses showing longer neurites, and formation of denser and more intricate neurite networks in miR-451a overexpressing cells at earlier time points than controls. Opposite changes were observed in NT2 cells with lentiviral knockdown of miR-451a expression. These findings were, furthermore, augmented by the detection of an imbalance between proliferation and differentiation of neural stem cells (NSC) in the brains of miR-451a^-/-^ mice indicating a possible role of miR-451a in neuronal differentiation *in vitro* and *in vivo*.

## Material and methods

### Cell culture and differentiation of NT2 cells

Ntera2/D1 (NT2) (ATCC, LCG standards) and HEK293T cells (human embryonic kidney cells, provided by Dr Alexander Deutsch, Division of Haematology, Medical University Graz) were maintained in high glucose Dulbecco’s modified Eagle Medium (DMEM, Sigma-Aldrich) containing 10% Fetal bovine serum (FBS, Sigma-Aldrich), 1% Non-Essential Amino Acids (NEAA, Sigma-Aldrich) and 1% Penicillin-Streptomycin (Sigma-Aldrich) at 5% CO_2_ and 37°C.

Differentiation of NT2 cells was performed as described before [14]. Briefly, 5x10^6^ undifferentiated NT2 cells were seeded into ultra-low attachment (ULA) flasks (VWR International) with 10 μM retinoic acid (RA, Sigma-Aldrich). Medium was changed every 2 days until day 15 and 10 μM RA was added. On day 15, medium was changed without adding RA. On day 17, neurospheres were plated onto flasks coated with a reduced growth factor basement membrane extract (Geltrex, Life Technologies) and cultivated in the presence of a mitosis inhibitory mixture (10 μM 5-Fluoro-2-Desoxyuridine, 1 μM Cytosine-β-D-Arabinofuranoside and 10 μM Uridine, Sigma-Aldrich) until day 28 with medium changes on alternating days.

### Transformation, transfection, determination of viral titre, transduction and FACS

Transformation with lentiviral vectors (AB.G.miR-451a, control vector AB.G.ct, G-U6-451PT and control vector G-0, kindly provided by Dr Papapetrou, Icahn School of Medicine in Mount Sinai, New York [15]) was done with GCI-5α or GCl-L3 super-competent *Escherichia coli* strains (THP Medical Products) according to the manufacturer’s instructions. Minipreps and maxipreps were performed according to the manufacturer’s instructions (Qiagen). Plasmid identities were confirmed by restriction enzyme digestion by incubating 500 ng of each plasmid with *Eco*RI and *Xho*I restriction enzymes for 1h at 37°C and subsequent analysis on a 2% agarose gel.

For production of lentiviral particles, 5x10^6^ HEK293T cells were seeded in a 10 cm dish (Szabo Scandic), and transfected at 80–90% confluency with lentiviral vector DNA using 3^rd^ generation packaging mix and Lentifectin reagent (ABM good) according to the manufacturer’s instructions. The pooled supernatant of two harvests was centrifuged at 3000 rpm for 15 min at 4°C. The cleared virus-containing supernatant was then filtered using low-protein binding 0.45 μm sterile filters (VWR International) and stored in aliquots at -80°C. Viral titre was determined using the Lenti-x p24 Rapid Titer Kit according to the manufacturer’s instructions (Clontech).

For transduction, 5x10^5^ NT2 cells were seeded in 75cm^2^ flasks and incubated at 37°C temperature with 5% CO_2_ until 30–50% confluent. Transduction was performed using 10^8^ viral particles/ml (multiplicity of infection (MOI) = 80; calculated as $MOI=\frac{volume\;of\;viral\;stock\times virus\;concentration}{volume\;of\;cell\;culture\times cell\;number\;in\;culture}$) and 6 μg/ml Polybrene (ABM good) on 3 consecutive days. Appearance of eGFP fluorescence was checked with a fluorescence microscope (Olympus) one or two days after the third transduction.

Fluorescence activated cell sorting was done with a FACS Aria IIu (BD Biosciences) by the Flow Cytometry Core Facility (Centre for Medical Research, Medical University of Graz). eGFP fluorescence was detected with a filter for FITC (530/30 BP) following excitation at 488 nm wavelength. eGFP^-^ NT2 cells were used for set up of the basic adjustments. Transduced cells were sorted in a 4-Way sort (maximal purity) into low, middle and high eGFP^+^ cells. Cell aggregates were excluded to prevent sorting of false positive cells (Sort adjustments: 100 μM Nozzle, pressure: 20 psi, frequency: 27 kHz, flow rate: 1–2, 4-Way sort (maximal purity).

### RNA, miRNA and protein isolation

Cell pellets were taken at different time points of neuronal differentiation (0, 8, 17, 22 and 28 days). RNA, miRNA and proteins were isolated from the same pellet using mirVana Paris Kit (Applied Biosystems) following the manufacturer’s protocol. For normalisation, lysis buffer was supplemented with RNA spike-in mix I (UniSp2, UniSp4 and UniSp5, Exiqon). RNA and miRNA concentrations were determined with a biophotometer (Eppendorf). RNA quality was checked on a denaturing RNA formaldehyde gel (1.2% agarose, 80 V, 35 min).

### cDNA synthesis, qPCR and data normalisation

cDNA synthesis from RNA was done with a RevertAid First Strand cDNA synthesis Kit (Thermo Fisher Scientific Bioscience) according to the manufacturer’s instructions (100 ng RNA per reaction). Spike-in was used for normalization (Tataa Biocenter).

cDNA synthesis from miRNA was performed with a miRCURY LNA Universal cDNA Synthesis Kit II (Exiqon) according to the manufacturer’s instructions (20 ng miRNA). For data normalisation, RNA spike-in mix II (UniSp6 and cel-miR-39-3p, Exiqon) was used. qPCR was done with a KAPA SYBR FAST LightCycler 480 kit (VWR International) in a LightCycler480 device (Roche Applied Science; miRNA PCR program: 1) Pre-incubation: 95°C, 10min, 2) Amplification: 45 cycles: a) Denaturation at 95°C, 10s; b) Annealing and Extension at 60°C 1min); mRNA PCR programme: 1) Pre-incubation: 95°C, 5min, 2) Amplification: 45 cycles: a) Denaturation at 95°C, 30s; b) Annealing at 60°C, 30s; c) Extension at 72°C, 30s). Negative controls were included in each experiment. Melting curve analysis was done according to the LC480 instruction manual (Roche Applied Science). qPCR reaction for each cDNA library was run in quadruplets. Relative mRNA expression was evaluated by using the 2^-ΔΔCt^ method without PCR efficiency correction [16]. Briefly, expression data was first normalized against a spike-in reference (ΔCt = Ct_Target_-Ct_Spike-in_) for each group and time-point separately, all of which were then normalized against the respective control vector group (AB.G.ct or G-0) group at day 0 of differentiation (ΔΔCt = ΔCt_Experiment_-ΔCt_ControlVector-day0_). Similarly, miR-451a target gene expression in G-0 and G-U6-451PT transduced cells at day 0 and day 22 were analysed with the same method, by normalisation against the G-0 group at the consistent time point (day 0 or day 22) (ΔCt = Ct_Target_-Ct_Spike-in_; ΔΔCt = ΔCt_Experiment_-ΔCt_G-0_). Fold-change of expression was obtained by calculating *log2*(2^-ΔΔCt^). The data is presented as the mean value of fold-changes ± SEMs obtained from three biological replicates. Primers for qPCR were found in the literature and ordered from companies (Origene; Exiqon; Primerdesign) or were designed using Primer3Output and then ordered from Eurogentec (**S1 Table**).

### Standard curves for absolute quantification of miRNA

A standard curve using defined copy numbers of mimics of miR-451a (Ambion mirVana miR-451a mimic, AAACCGUUACCAUUACUGAUU) was created using qRT-PCR. The molecular weight and starting copy number/μl were calculated. Serial dilutions were prepared, starting with undiluted mimics (10^13^ molecules/μl) including 7 dilutions and converted into cDNA using miRNA Universal cDNA Synthesis Kit II from Exiqon. qPCR was done as described above. Standard curves were designed by plotting the measured Ct-values on the y-axis against natural logarithmic (ln) values of the specific copy number of mimic used as described earlier [17]. A trend line was plotted through the data points and the resulting formula was used to calculate the copy number per 20 ng used miRNA using Microsoft Excel 2010 (Microsoft Corporation).

The copy number/20 ng miRNA was normalized by multiplication with a normalisation factor [17] generated using spike-in before cDNA synthesis (Exiqon).

### Western blot

Cells were harvested and immediately homogenized using mirVana Paris Kit (Applied Biosystems) following the manufacturer’s protocol. Protein content was determined using BCA Protein Assay Kit (Novagen). 30 μg of protein was loaded on a 10% SDS-PAGE gel followed by transfer to nitrocellulose (Amersham Protran 0.45μm NC; GE Healthcare Life Science) in a Tank Blotter (Biorad). Blots were blocked with StartingBlock T20 (TBS) Blocking Buffer (Thermo Fisher Scientific) for 1 h. Filters with specific antibody solutions were incubated overnight in blocking solution (Rabbit anti TNS4 1:500; Abcam), followed by 2x TBST and 3x TBS washing steps and incubation with biotinylated antibody solutions for 1 h. After TNS4 visualisation the membrane was stripped using Restore Western Blot Stripping buffer (Thermo Fisher Scientific) for 30 min, blocked and incubated overnight in mouse anti β-Tubulin (1:5000; Sigma) followed by the procedure described above. For visualization, blots were washed again and developed by SuperSignal West Pico PLUS Chemiluminescent Substrate (Thermo Fisher Scientific).

### Ethics statement and animal husbandry

Experimental animals were cared for and used in accordance with ethical guidelines and all animal experiments were approved by the Bundesministerium für Wissenschaft, Forschung und Wirtschaft (BMWF-66.010/0100-II/3b/2013). Wild type (C57BL/6N mice, Charles River Labs) and miR-451a^-/-^ mice were kept under standard conditions with food and water *ad libitum* in a controlled environment with a 12h:12 h light-dark cycle, in the animal facility of the Biomedical Research Institute at the Medical University of Graz.

### Preparation of tissue samples for immunofluorescence

Mice were euthanized at postnatal days 5, 15, 25, 30, 35, 40 and 50 via i.p. injection of ~10 ml/kg body weight Thiopental Sodium (Sandoz) (50 mg/ml in physiological saline) and transcardially perfused with 4% formalin prepared from a 37% stock (Merck) in phosphate buffered saline (PBS, pH 7.4) (Sigma-Aldrich). Brains were removed and stored in 4% formalin overnight at 4°C. Following fixation, brains were incubated in 20% sucrose for cryo-preservation and tissue slices were prepared as frozen coronal sections (thickness 20 μm).

### Immunofluorescence of cells and microscopy

Neurospheres from non-transduced and transduced cells were plated on day 17 on Geltrex (Life technologies) coated glass slides (Thermo Fisher Scientific) in 6 well plates (VWR International) and differentiated as described above. Cells were fixed with 4% Paraformaldehyde-PBS (VWR International) for 20 min at various differentiation stages, and then stained by indirect immunofluorescence using a standard protocol. Briefly, fixed cells were washed 3 times for 5 min with 1x PBS, incubated for 30 min in 0.3% Triton X-100-PBS (Sigma-Aldrich), washed 3 times for 5 min in 1x PBS and then blocked in 1% BSA-PBS solution for 60 min at RT.

The incubation with the primary antibody (chicken anti-NF200 (1:500, Abcam), chicken anti-MAP2 (1:1000, Abcam), goat anti-Doublecortin (1:250, Santa Cruz), chicken anti-βIII Tubulin (1:1000, Abcam) and mouse anti-TNS4 (1:250, Abcam)) was done overnight at 4°C. The cells were washed 3 times for 5 min with 1x PBS before application of the respective secondary antibody (Rabbit anti chicken FITC, Donkey anti goat FITC or Goat anti mouse FITC, all from Abcam and 1:2000) for 1h at RT. After two 5 min washing steps with 1x PBS the cells were covered with Fluoroshield, a DAPI-containing aqueous mounting media (Sigma-Aldrich).

Cells were analysed with a fluorescence microscope (Olympus, used objective: 20x), a Zeiss Laser Scanning Microscope (LSM 510 META, Zeiss; integrated lasers are UV 405nm, multiline Argon 458/477/488/514nm, Helium-Neon 543nm as well as Argon 514 and Helium-Neon 633nm, used objective: EC Plan-Neo 40x/1.3 Oil with DIC capability) or an Ultra-Fast Spectral Scanning Confocal Microscope (Nikon A1R, Nikon, integrated lasers are Violet diode 405nm, multiline Argon 457-514nm, DPSS laser 561nm, Diode Laser system 642nm, used objective: CFI Plan Apochromat Lambda 40x/0.95).

### Immunofluorescence of tissue samples and microscopy

Tissue sections were incubated in 4% formalin in PBS for 10 min for post-fixation. Antigen retrieval was done using sodium citrate buffer pH 6.0 (10mM C_6_H_5_Na_3_O_7_ x 2H_2_O (Sigma-Aldrich), 0.05% Tween-20 (VWR, Leuven, Belgium) in pure water) for 3 min at 90–100°C. Sections were later incubated in 2N hydrochloric acid (HCl) (Merck) for 30 min and in borate buffer pH 9.0 for 6 min (50 mM BH3O3 (Sigma-Aldrich), 5 mM Na_2_B_4_O_7_ x10H_2_O (Sigma-Aldrich), 10 mM Na_2_SO_4_ (Sigma-Aldrich). Blocking was performed for 1 hour at room temperature (10% Normal Goat Serum (Sigma-Aldrich), 0.3% Triton X-100 (Sigma-Aldrich) in PBS) to avoid non-specific antibody binding. For Ki67 detection, overnight incubation with rabbit monoclonal anti-Ki67 (1:500; Abcam) at +4°C was used. Doublecortin was detected using a chicken polyclonal anti-doublecortin (1:500; Abcam) incubated overnight at +4°C. Secondary antibodies were 1:1000 goat anti-rabbit IgG conjugated to Alexa488 fluorophore (Abcam) and 1:1000 goat polyclonal anti-chicken IgY conjugated to Alexa647 fluorophore (Abcam). For counterstaining and mounting, an aqueous mounting media containing 4',6-diamidino-2-phenylindole (Fluoroshield with DAPI (Sigma-Aldrich) was utilized. Microscopy was performed using Axio Imager 2.1 (Carl Zeiss AG, Oberkochen, Germany) installed with TissueFaxs Cell Analysis System (TissueGnostics GmbH, Vienna, Austria). Images were taken at 20X magnification.

### In situ hybridization

4% PFA fixed, paraffin-embedded brains of male wild type C57BL/6N mice at post-natal day 5, day 15, and adult stage (Charles River Labs) were sectioned at 5μm thickness for the analysis of miR-451a expression. All probes and the miRCURY LNA microRNA ISH optimization Kit (FFPE) were purchased from Exiqon. Sections were deparaffinized in xylene, rehydrated with an ethanol gradient, and treated with 2 μg/mL proteinase K (Roche Diagnostics) for 10 min at 37°C. Hybridization was performed overnight at 45°C with 25 nM double-Digoxigenin (DIG) custom miRCURY LNA probe for miR-451a (Sequence: 5’-AACTCAGTAATGGTAACGGTTT-3’), and scrambled probe (Sequence: 5’-GGTAGTATATTAATAAGCCCTG-3’). Sections were then stringently washed at 45°C with SSC solution according to the manufacturer’s instructions, blocked for 1 h with 2% blocking solution and then incubated with sheep anti-DIG-AP Fab fragments at 1:800 (Roche Diagnostics) for 3 h at room temperature. Following three washes with 0.1% Tween PBS (PBS-T) pH 7.4 for 3 min each, the miRNA signal was detected with 5-bromo-4-chloro-3-indolyl phosphate (BCIP) and nitroblue tetrazolium (NBT) substrates (NBT/BCIP stock solution, Roche Diagnostics) in B1 (0.1 M Tris-HCl pH 9.5/0.1 M NaCl/50 mM MgCl_2_) for 24 h at 30°C. Staining was terminated by two 5 min washes with KTBT buffer (50 mM Tris-HCl, 150 mM NaCl, and 10 mM KCl), and by briefly rinsing twice in ddH_2_O before mounting sections with Kaiser’s glycerol gelatine. A Leica DM4000 B microscope (Leica Cambridge Ltd) equipped with Leica DFC 320 Video camera (Leica Cambridge Ltd) was used to acquire and analyse computerized of section images.

### Light microscopy and live imaging

All transduced cells were observed with a microscope (Olympus). Pictures were taken of different passages of transduced cells. Differentiating cells were photographed at different time points using a microscope (Olympus). Living cells were observed with a CellIQ (Cell IQ V2 MLF Cell Imaging and Analysis System, Imagen, Massachusetts, USA). Differentiating neuronal precursor cells were seeded into 6-well plates at day 17 with a mitosis inhibitor mix. Cells were then observed for 10 consecutive days with medium changes and addition of mitosis inhibitor mix every 2 days.

### Data analysis

Expression data was analysed with Microsoft Excel 2010 (Microsoft Corporation), IBM SPSS Statistics 22 and GenEx (MultiD Analyses AB).

Pictures obtained by immunofluorescence staining were analysed with the plugin NeuronJ [18–20] within the program ImageJ (NIH, USA) [18, 21]. Soma sizes in TNS4 immunostainings and [*neurite length/neurosphere diameter*] ratios were analysed using Imaris software (Bitplane). For NF200 immunostained samples, neurite length was measured using ImageJ. As the number of biological replicates was only 3 for qRT-PCR expression data, we performed non-parametric statistical tests. Friedman’s test was used to compare gene expression at different time points of differentiation within the mRNA or miRNA expression profile. Statistical significance of differences in neurite length after NF200 immunostainings was tested using Kruskal-Wallis H Test with Bonferroni correction in pairwise analyses. The data for [*neurite length/neurosphere diameter*] ratios were presented as percentage of the respective control group in overexpression and knockdown experiments (% of AB.G.ct and G-0, respectively) and the statistical significance of differences were tested with t-test. For these analyses normal distribution and variance homogeneity were confirmed using Wilk-Shapiro and Levene’s tests, respectively. Statistical significance of differences in mean cell soma areas and mean fluorescence intensity in the TNS4-stained cells at day 22 of differentiation was tested with Welch’s variance-weighted ANOVA due to the variance heterogeneity detected with the Levene’s Test. Games-Howell test was used for post-hoc comparisons in these analyses. The Mann-Whitney U-Test was used to detect significant differences between AB.G.ct cells and transduced AB.G.miR-451a cells. Ki67^+^ cells in the subgranular zone (SGZ) of dentate gyrus (DG) in hippocampus and subventricular zone (SVZ) of lateral ventricles were counted using ImageJ 1.46r [21]. Each group (wild type or miR-451a^-/-^) comprised *n≥4* animals per age. At least 2 coronal sections from each animal were used for quantification, resulting in 4 cerebral hemispheres sections. The total number of Ki67^+^ cells in SGZ and SVZ from one hemisphere of a coronal section was taken as “the total number per hemisphere”. For one animal, “mean total number per hemisphere” was calculated as the mean value of Ki67^+^ cells from these 4 hemispheres and this value was used as one data point in the final analysis. The statistical significance of the differences in the number of Ki67^+^ cells in the brains of wild type and miR-451a^-/-^ mice was assessed with the Mann-Whitney U Test. Researchers performing the microscopic evaluations and all quantifications were blinded with respect to the experimental groups.

A difference with *p≤0*.*05* was deemed statistically significant in all statistical assessments. All statistical analyses were carried out using SPSS v. 22/23/25 (IBM, USA).

## Results

### Endogenous miR-451a expression is upregulated during neuronal differentiation of Ntera2/D1 cells

**Fig 1:**
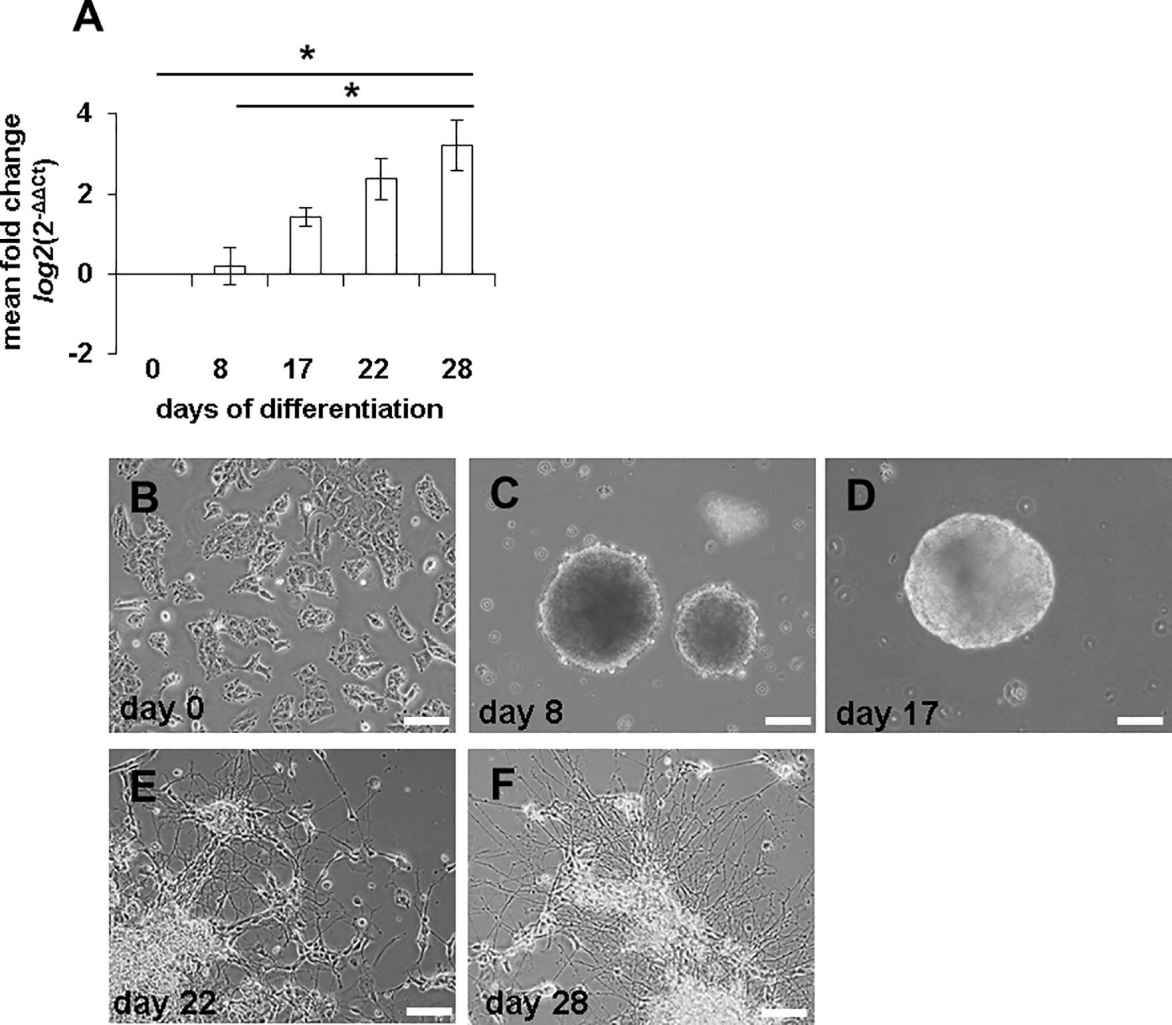
Endogenous miR-451a expression increases during neuronal differentiation. Endogenous miR-451a expression was upregulated time-dependently during neuronal differentiation of NT2 cells (**A**). (**B-F)** show neuronal microscopic images of NT2 cells during retinoic acid-induced differentiation in vitro. The statistical significance of changes in miR-451a expression was tested using Friedman‘s test. *p≤0.05 after Bonferroni correction. *n = 4* biological replicates; Scale bars: 100 μm. Error bars show standard error of the mean (SEM).

We first analysed endogenous miR-451a expression during neuronal differentiation of NTera2/D1 (NT2) cells. Endogenous miR-451a expression at day 28 of neuronal differentiation was three-fold higher than day 0 levels (**Fig 1A**). Increased miR-451a expression, particularly during late neuronal differentiation, moreover correlated with prominent neurite outgrowth and network formation at day 22 and day 28 (**Fig 1B–1F**) suggesting a role for miR-451a in neuronal differentiation.

### MiR-451a overexpression shifts mRNA expression of neuronal differentiation markers to earlier time points

To further assess a role of miR-451a in neuronal differentiation, undifferentiated NT2 cells were transduced with a lentiviral miR-451a overexpression vector (AB.G.miR-451a) or a control vector (AB.G.ct) [15]. In both groups, transduced cells were sorted according to the magnitude of their eGFP fluorescence (**S1 Fig**). Cells with high eGFP fluorescence were subsequently cultivated for RA-induced *in vitro* neuronal differentiation.

**Fig 2:**
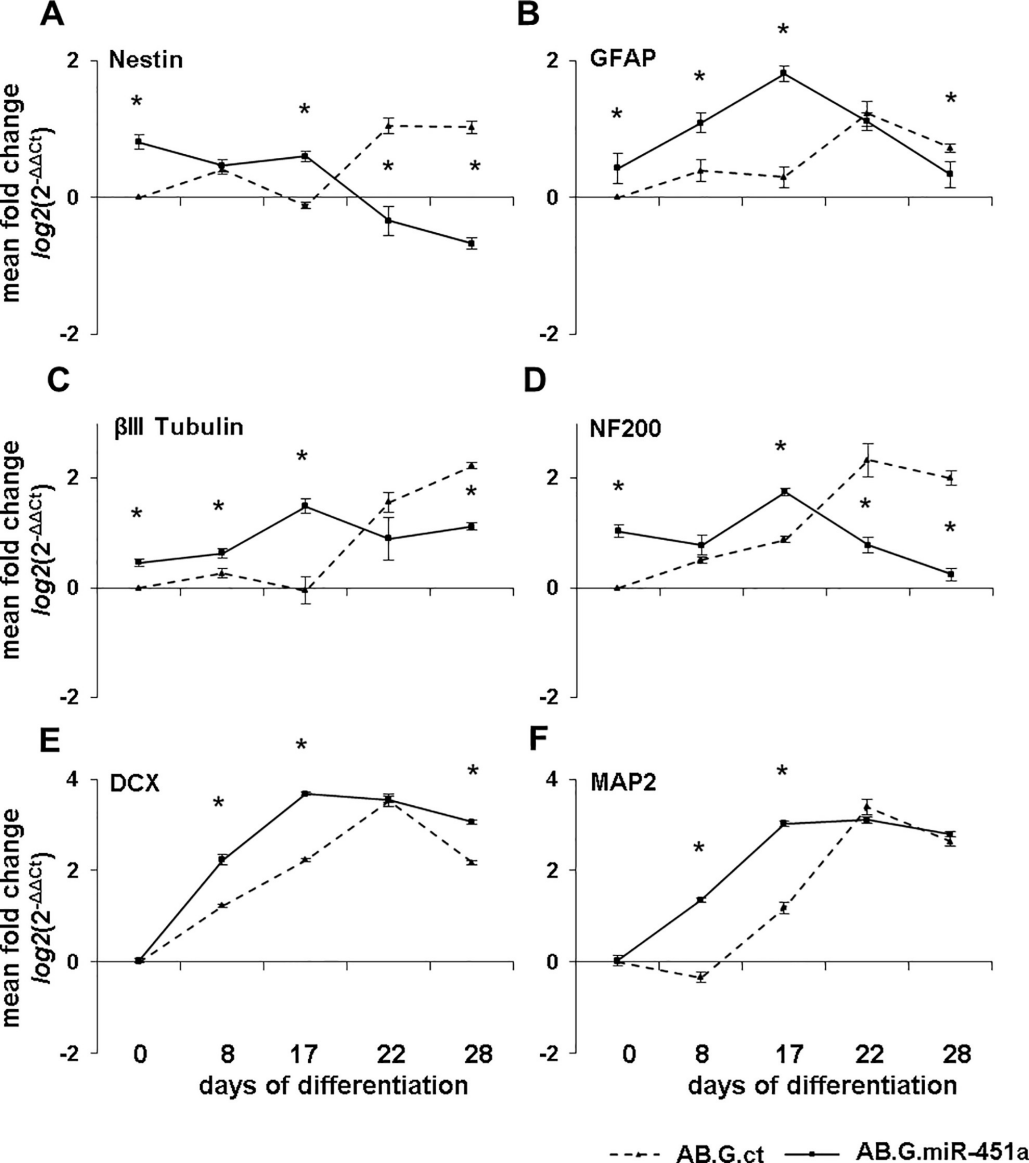
Differential expression of neural precursor and neuronal differentiation markers upon miR-451a overexpression. Expression of neural stem/precursor cell marker Nestin (**A**), was downregulated at days 22 and 28 in AB.G.miR-451a transduced cells, compared to control cells. Expression profiles of neuronal differentiation markers GFAP (**B**), βIII Tubulin (**C**), NF200 (**D**), DCX (**E**) and MAP2 (**F**) were shifted significantly to an earlier developmental time point in AB.G.miR-451a transduced cells compared to the control (AB.G.ct). The statistical significance of the observed changes was assessed using the Mann-Whitney U-test. *p≤0.05. *n = 3* biological replicates. Error bars show standard error of the mean (SEM).

qRT-PCR-based comparison of neural precursor and neuronal differentiation marker expression in NT2 cells transduced with AB.G.miR-451a and AB.G.ct vectors on day 0, 8, 17, 22 and 28 of RA-induced differentiation revealed striking differences (**Fig 2**).

mRNA expression of Nestin, an early neural stem/precursor cell marker, was significantly elevated in AB.G.miR-451a transduced, undifferentiated cells and this elevated expression maintained up to day 17. At days 22 and 28, however, Nestin expression was strongly downregulated in these cells as expected at later stages of differentiation [22, 23]. In contrast, Nestin expression increased in AB.G.ct cells at days 22 and 28 of differentiation (**Fig 2A**) suggesting a higher percentage of neural stem or neuronal precursor cells in these neurospheres at days 22 and 28, whilst most of the AB.G.miR-451a transduced cells were at the post-mitotic stage at these time points, in line with morphological observations.

Glial fibrillary acidic protein (GFAP) is known to be a marker of radial glial cells that differentiate from neuroepithelial cells and further give rise to both glial and neuronal lineage [24], and known to be upregulated during RA-induced differentiation of NT2 cells [25–28]. Consistent with this, a similar transient increase was observed in our setting, both with AB.G.miR-451a and AB.G.ct transduced cells (**Fig 2B**). GFAP upregulation was, however, significantly higher in AG.B.miR-451a cells at day 0, 8 and 17, compared to control cells. Peak GFAP expression was, furthermore, observed at day 17 for AB.G.miR-451a transduced cells, compared to day 22 for AB.G.ct transduced cells.

mRNA expression of βIII Tubulin, an early neuronal marker, was significantly upregulated in AB.G.miR-451a cells at days 0, 8 and 17 (**Fig 2C**), whilst in AB.G.ct cells βIII Tubulin expression was upregulated starting at day 22.

Neurofilament 200 (NF200) proteins are early to intermediate neuronal cell markers [29, 30]. mRNA expression of NF200 was significantly higher in AB.G.miR-451a transduced cells up to day 17 than in controls, but showed a marked decrease at days 22 and 28. In AB.G.ct transduced cells, in contrast, NF200 expression increased during neuronal differentiation until day 22 (**Fig 2D**).

Doublecortin (DCX), a marker for neuronal precursor cells and immature neurons, was upregulated during neuronal differentiation of both AB.G.ct and AB.G.miR-451a transduced cells. Decreased DCX expression was observed in AB.G.miR-451a transduced cells from day 22, indicating neuronal maturation, whilst a similar reduction occurred in AB.G.ct cells from day 28 (**Fig 2E**).

Microtubule-associated protein 2 (MAP2) is a neuron-specific cytoskeletal protein that is enriched in dendrites and has been implicated to play a role in determining and stabilizing dendritic shape during neuronal development [31, 32]. MAP2 was significantly upregulated in AB.G.miR-451a transduced cells from day 8 onward, whilst AB.G.ct cells exhibited upregulation later, at day 17 (**Fig 2F**). Peak MAP2 mRNA expression was reached in AB.G.miR-451a transduced cells at day 17 and in AB.G.ct cells at day 22.

Three of these differentiation markers, Nestin, βIII Tubulin and NF200, were analysed at day 0 and day 22 of differentiation in G-U6-451PT (miR-451a silencing) and G-0 (control vector) transduced cells. In contrast with the elevated levels of these markers at day 0 upon miR-451a overexpression, downregulations were observed upon miR-451a silencing as compared to the controls, although the differences were statistically not significant. At day 22, however, mRNA expression of all three markers showed 4-to-6 fold increases in both G-U6-451PT and G-0 transduced cells, similar to the upregulations observed in AB.G.ct controls (**S2 Fig**).

Taken together, these data are consistent with accelerated neuronal differentiation of miR-451a overexpressing cells.

### MiR-451a overexpression accelerates neurite outgrowth and network formation

**Fig 3:**
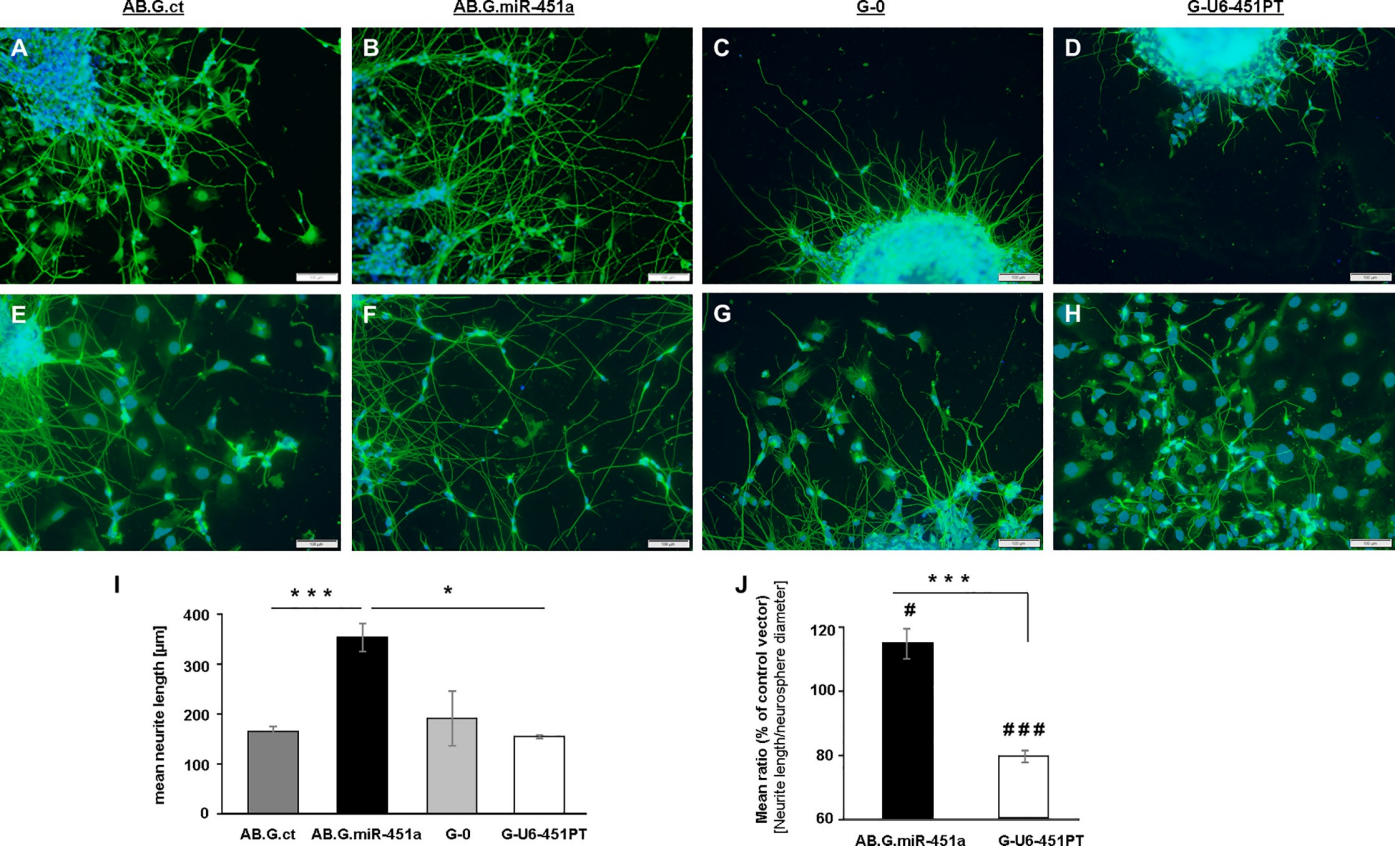
MiR-451a overexpression promotes neurite outgrowth from neurospheres. AB.G.ct neurospheres showed less neurite outgrowth from adherent neurospheres at day 22 of differentiation (**A**) as compared to AB.G.miR-451a neurospheres (**B**). G-0 neurospheres (G-0: control for miR-451a silencing vector) (**C**) showed more neurite outgrowth than G-U6-451PT neurospheres (miR-451a silenced) (**D**). Most branched and densest neurite networks with longer neurites were observed in AB.G.miR-451a neurospheres (**E-H**). On the other hand, remarkably shorter neurites and lower network density were observed upon miR-451a knockdown (**D, H**). Neurospheres were immunostained for Neurofilament heavy chain (NF200). Quantification of mean neurite length (*n≥3* neurospheres per group) (**I**) confirmed the statistical significance of longer neurite observation in miR-451a overexpressing neurospheres. The mean ratio of the length of single neurites to neurosphere diameter at day 22 of differentiation in AB.G.miR-451a and G-U6-451PT transduced cells (**J**) (*n≥8* neurospheres per group) further confirmed the effect of miR-451a in neurite outgrowth from differentiating neurospheres. Statistical significance of the differences were tested by Kruskal-Wallis Test with Bonferroni correction for post-hoc analyses (**I**) or by t-test following confirmation of normal distribution with Wilk-Shapiro test (**J**). *p<0.05, ***p<0.001 in between compared groups; ^#^p<0.05, ^###^p<0.001 as compared to the respective control vectors (AB.G.ct or G-0). Error bars represent standard error of the mean (SEM). Scale bars: 100 μm.

Seeding of neurospheres on plates coated with reduced growth factor basement membrane extract was followed by cell migration out of the neurosphere, the formation of neurites and the start of network formation. Analyses of NF200^+^ neurites by immunohistochemistry at day 22 revealed longer neurite outgrowth from AB.G.miR-451a transduced cells than from AB.G.ct cells (**Fig 3A and 3B**). On the other hand, G-U6-451PT transduced cells exhibited much shorter neurites as compared to both G-0 and AB.G.miR-451a group (**Fig 3B–3D**). Qualitative assessment of migrant cells from adherent neurospheres revealed network formation by AB.G.miR-451a transduced cells remarkably more than by controls cells (**Fig 3E and 3F**), while in G-U6-451PT transduced cells network formation was observed to be much less than the respective control group (G-0) and AB.G.miR-451a transduced cells (**Fig 3F–3H**). The statistical significance of these differences was further confirmed by quantitative analysis of average neurite length (AB.G.ct 164.25 ± 10.93 μm, AB.G.miR-451a: 353.21 ± 27.75 μm; G-0: 190.69 ± 54.86 μm; G-U6-451PT: 153.68 ± 3.54 μm). A Kruskal-Wallis H test showed that there was a statistically significant effect of miR-451a expression on average neurite length at day 22 of differentiation (χ^2^(3) = 22.943, p<0.001 with mean rank neurite lengths: AB.G.ct: 11.64, AB.G.-miR-451a: 28.75, G-0: 12.67 and G-U6-451PT: 10.50). Pairwise comparisons revealed that the neurites in AB.G.miR-451a transduced cells were significantly longer than AB.G.ct group (p<0.001) and G-U6-451PT group (p = 0.015) (**Fig 3I**). Further, there was a statistically significant increase in average ratio of [*neurite length/neurosphere diameter*] at day 22 in AB.G.miR-451a transduced cells (mean = 114.79%, standard deviation (SD) = 14.77) as compared to the AB.G.ct group (mean = 100%, SD = 12.89) (t(19) = -2.450, *p = 0*.*024*). On the contrary, a statistically significant decrease was detected in average ratios for G-U6-451PT transduced group (mean = 79.22%, SD = 5.18) as compared to the G-0 group at day 22 of differentiation (mean = 100%, SD = 4.97)) t(14) = 8.177, *p<0*.*001*). Ratio of [*neurite length/neurosphere diameter*] in AB.G.miR-451a transduced cells was also significantly higher than that of G-U6-451PT transduced group (t(11) = 7.087, *p<0*.*001*) (**Fig 3J**). Although, there was a marked increase in network formation in AB.G.ct cells between days 22 and 28, the qualitative differences were observed to be sustained at day 28, by which time AB.G.miR-451a transduced cells had developed a much more intricate neurite network than AB.G.ct cells (**S3 Fig**).

**Fig 4:**
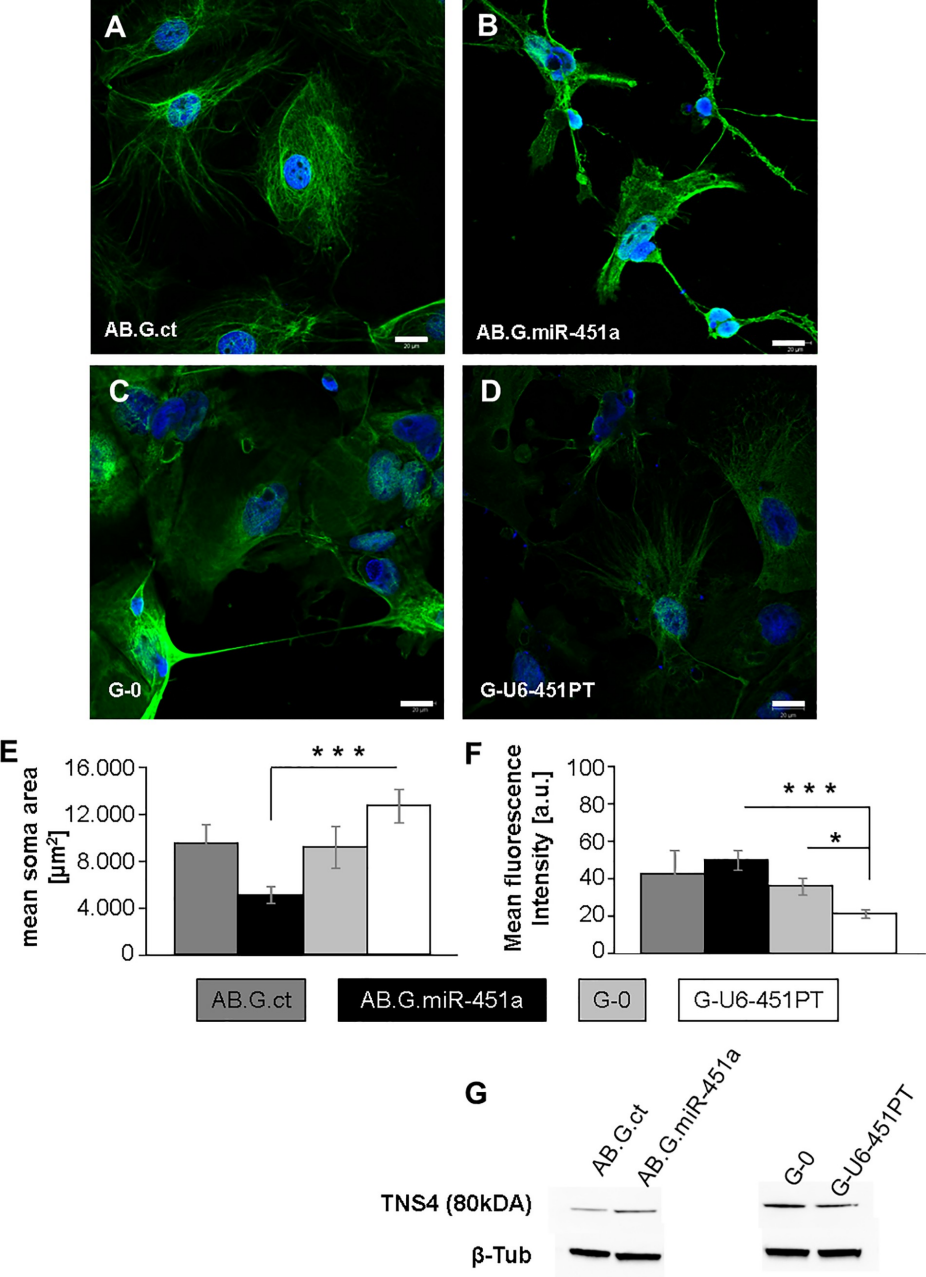
NT2 cells show different morphology upon miR-451a overexpression. MiR-451a overexpression resulted in a more compact cell shape with smaller soma size (**A, B**), while larger soma size was observed with miR-451 knock-down (**C, D**). Quantitative analysis of soma size in all groups of cells confirmed the significance of these observations (**E**). Mean fluorescence intensity of TNS4 fluorescent immunocytochemistry (ICC) in these cells showed significantly lower TNS4 expression in G-U6-451PT transduced group (miR-451a knock down) as compared to both AB.G.miR-451a (miR-451a overexpression) and G-0 (control vector) transduced groups (**F**). (**G**) shows a representative image of Western blot (WB) for TNS4 protein expression in all groups with β-Tubulin as internal control. ICC and WB pictures are representative of at least 3 different experiments. The statistical significance of the differences was assessed with Welch’s variance-weighted ANOVA followed by Games Howell test for post-hoc analysis (n≥6 neurospheres per group). Error bars show standard error of the mean (SEM). *p≤0.05, ***p≤0.001. Scale bars: 20 μm.

In order to further assess neuronal maturation and migratory potential, Tensin-4 (TNS4) staining was utilized (**Fig 4**). TNS4 is a focal adhesion protein that promotes cell migration by triggering the uncoupling of integrins from the actin cytoskeleton [33–35].

In all positively stained cells neurite outgrowth and network formation was accompanied by morphological changes. Upon neuronal maturation, cells change from a spherical to a bipolar phenotype with a shrunken soma. Migration out of the neurosphere was furthermore observed. At day 22 of differentiation, miR-451a overexpression (AB.G.miR-451a) resulted in a rather compact cell size with shrunken soma as compared to the control group (AB.G.ct) (**Fig 4A and 4B**), whilst larger soma size was observed in cells with miR-451a knockdown (G-U6-451PT) as compared to both respective control group (G-0) and miR-451a overexpressing cells (**Fig 4B–4D**). Quantitative analysis revealed a statistically significant effect of miR-451a expression on cell soma size (F[3,18] = 9,063, *p = 0*.*001*), where miR-451a overexpressing cells had significantly smaller somas (5.072,94 ± 705,23 μm^2^) as compared to the cells with miR-451a knock-down (12.656,56 ± 1.438,42 μm^2^; *p<0*.*001*) (**Fig 4E**). Although AB.G.miR-451a transduced cells had remarkably smaller somas as compared to AB.G.ct cells (9.749,16 ± 1.591,04 μm^2^), the differences were statistically not significant (*p = 0*.*138*). Similarly, average soma area in miR-451a knock down cells tended to be larger than the respective control group (G-0: 11.320,31 ± 2.163,89 μm^2^), though statistically not significant.

TNS4 staining revealed diffuse expression in the soma of AB.G.ct cells (**Fig 4A**), whilst in AB.G.miR-451a cells, stronger TNS4 staining was observed in neurites (**Fig 4B**). In contrast, a much more diffuse expression of TNS4 was observed in G-U6-451PT cells as compared to G-0 or AB.G.miR-451a groups (**Fig 4B–4D**). Next, we quantified the mean fluorescence intensity to assess whether the observed differences point to a differential expression of TNS4 upon overexpression or knock-down of miR-451a in differentiating cells at day 22. Indeed, a statistically significant difference was observed in the mean fluorescence intensities of TNS4 stainings (F[3,16] = 9,374, *p = 0*.*001*). In detail, there was a robust decrease of TNS4 signal intensity in G-U6-451PT transduced cells (21,16 ± 2,23) as compared to both G-0 transduced cells (35,94 ± 4,53; *p<0*.*05*) and AB.G.miR-451a transduced cells (49,74 ± 5,33; *p<0*.*001*) (**Fig 4F**). Increases in the signal intensity were observed in AB.G.miR-451a transduced cells compared to AB.G.ct cells (42,52 ± 12,59), but the difference was statistically not significant. Nevertheless, a slight increase in TNS4 protein expression in miR-451a overexpressing cells compared to control group (AB.G.ct) as well as the reduction in miR-451a knock-down group as compared to consistent control group (G-0) were confirmed with Western blots (**Fig 4G**).

These results suggest that miR-451a overexpression of induces neuronal maturation of cells, followed by accelerated growth of neurites and network formation during neuronal differentiation.

### MiR-451a targets

In order to assess how miR-451a modulates neuronal differentiation, mRNA expression of validated and predicted miR-451a targets was analysed by qRT-PCR during neuronal differentiation of miR-451a overexpressing and control cells. For this purpose, different target prediction programs and databases providing information on experimentally validated miRNA targets were utilized (**Table 1**).

**Table 1:** Information regarding selected target genes and queried databases.

Target Gene	Databases	Position of binding site*	mirSVR score*	Involvement in neurogenesis	Refs.
**PSMB8**^a^	miRanda, TargetScan, miRSearch, DIANA microT, TarBase	214 of 240	-1.2960	-	[36]
**CXCL16** ^a^	miRanda, miRSearch	225 of 968	-1.1813	-	[37]
**MIF** ^a^	miRanda, miRSearch, Diana MicroT, miRTarBase	90 of 116	-1.0769	[38, 39]	[40–42]
**CAB39** ^a^	miRanda, TargetScan, Diana MicroT, TarBase, miRTarBase	98 of 2370	-0.5777	-	[43–48]
**CDKN2D** ^a^	miRanda, TargetScan, miRDB	223 of 595	-0.5422	[49, 50]	[51]
**YWHAZ** ^a^	miRanda, TargetScan, miRDB	565 of 2117	-0.5074	[52]	[53–59]
**IL6R** ^a^	miRanda, TargetScan, miRTarBase	1) 905 of 2332 2) 2095 of 2332	-0.4786 -0.0094	-	[60]
**RAB14** ^a^	miRanda, Diana MicroT, TarBase, miRTarBase	1) 332 of 3258 2) 2658 of 3258 3) 3194 of 3258	-0.0093 -0.4533 -0.1247	-	[61, 62]
**TSC1** ^a^	miRanda, TargetScan, Diana MicroT	1) 138 of 4887 2) 558 of 4887 3) 2203 of 4887	-0.0545 -0.1106 -0.0003	[63]	[2, 64]
**AKT1** ^b^	TarBase, miRTarBase	NA	NA	[65]	[1, 66–68]
**OSR1** ^c^	miRanda, TargetScan, miRSearch, Diana MicroT, miRDB	758 of 789	-1.345	[69]	
**POU3F2** ^c^	miRanda, miRSearch,	1547 of 2584	-0.8820	[70]	
**TNS4** ^c^	miRanda,	1528 of 1758	-0.0092	-	

Information regarding selected target genes (validated or predicted), utilized databases and miR-451a binding positions on the transcripts. Notably, around half of the targets were shown to be involved in neurogenesis.

*Applicable to miRanda only.

^a^Validated

^b^Validated but no binding site exists

^c^Predicted.

Searches of miRTarBase 4.5 and DIANA-TarBase V7.0 returned 23 and 37 validated genes, respectively. Binding sites for selected genes were reviewed using miRanda to judge specificity, efficiency and stability of miR-451a binding. Based on this information, mRNA expression of validated targets PSMB8, CXCL16, MIF, CAB39, CDKN2D, YWHAZ, IL6R, RAB14, TSC1, AKT1 and predicted targets OSR1, POU3F2, TNS4 was analysed in mRNA from AB.G.ct and AB.G.miR-451a transduced cells. The mRNA profiles so obtained in essence revealed two modes of mRNA regulation in response to miR-451a overexpression.

**Fig 5:**
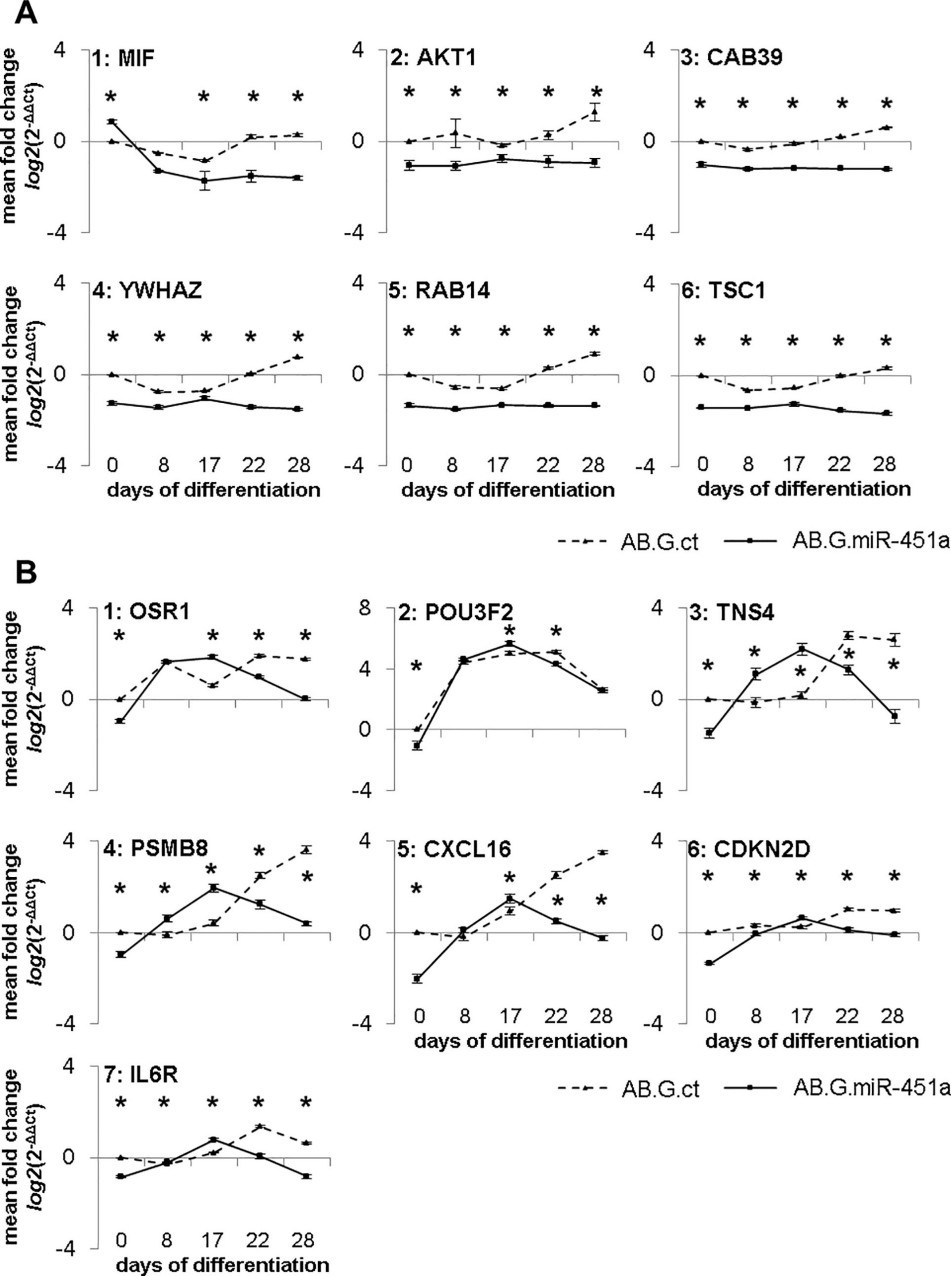
MRNA expression of miR-451a targets in AB.G.ct and AB.G.miR-451a transduced NT2 cells. mRNA expression of predicted and validated target genes of miR-451a differ remarkably in AB.G.ct and AB.G.miR-451a transduced cells during neuronal differentiation. (**A1-A6**) show target genes that were significantly downregulated in response to miR-451a overexpression. (**B1-B7**) show a second group of target genes that exhibited a modified expression profile upon miR-451a overexpression. The statistical significance of the observed differences was assessed with the Mann-Whitney U-test, *p≤0.05. *n = 3* biological replicates. Error bars show standard error of the mean (SEM).

In the first group consisting of MIF, AKT1, CAB39, YWHAZ, RAB14 and TSC1 mRNA, target expression was significantly lower in AB.G.miR-451a cells than in cells transduced with the control vector (**Fig 5A1-5**) at all time points analysed.

In the second group, consisting of OSR1, POU3F2, TNS4, PSMB8, CXCL16, CDKN2D and IL6R, mRNA expression in AB.G.miR-451a clearly followed a developmental expression, but peak mRNA expression was, in each case, shifted to earlier time points (**Fig 5B1-7**) and significant downregulation only observed at late differentiation stages. Therefore, contrary to our expectations, mRNA expression of these validated/predicted genes was not always downregulated, but instead was upregulated compared to AB.G.ct transduced cells.

Five of the verified targets of miR-451a, which were downregulated upon miR-451a overexpression (MIF, AKT, CAB39, YWHAZ and TSC1), were analysed for their expression levels in G-U6-451PT or G-0 transduced NT2 cells at day 0 and day 22 of RA-induced differentiation. All five of them showed upregulated expression upon miR-451a knock-down (G-U6-451PT) both at day 0 and day 22 compared to the G-0 group at the respective time points (**S4 Fig**), although the differences were statistically not significant.

### Endogenous miR-451a expression in vivo

**Fig 6:**
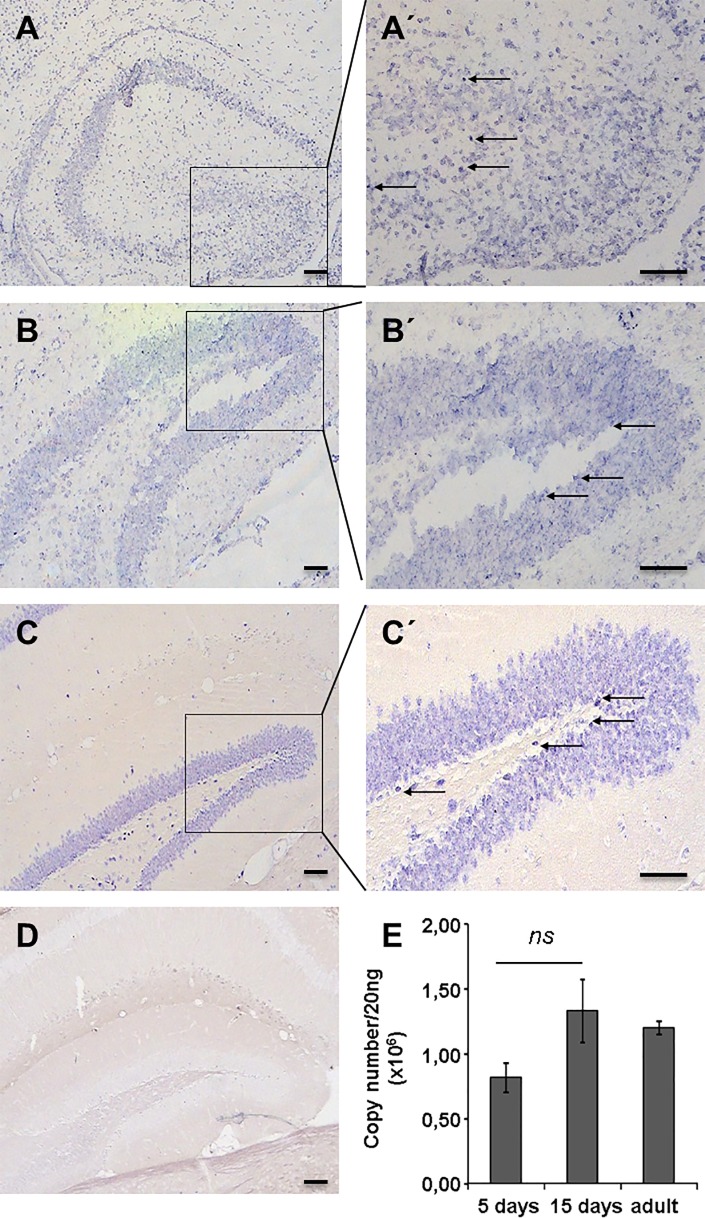
MiRNA-451a expression in hippocampus of wild type mice. miRNA-451a in situ hybridization was carried out on coronal brain sections of male C57BL/6N mice at postnatal day 5 (**A**), postnatal day 15 (**B**) and adult stage (**C**). (**A´, B´, C´**) show the respective regions at higher magnification. Intensively stained single cells were found predominantly in the subgranular zone and the hilus (**arrows**). (**D**) shows a hybridization control with a scrambled riboprobe. (**E**) shows quantitative analysis of miR-451a expression in hippocampal formation assessed by qPCR at postnatal day 5, day 15 and at the adult stage. In situ hybridizations were conducted with at least 15 coronal sections per animal with *n≥5* animals per group. qPCR analysis was conducted with *n = 3* animals per group with 8 qPCR replicates. ns: not significant. Scale bars: 100 μm.

To clarify a possible role of miR-451a in *in vivo* neuronal development and differentiation, the expression pattern of miR-451a in wild type mouse brains at different developmental stages was assessed by in situ hybridisation. MiR-451a expression was monitored at days 5, 15 and in adult hippocampus (**Fig 6A–6C**). MiR-451a expression was detected at all stages of postnatal hippocampal development. Neurons of the CA1-CA4 region, as well as granular cells of the dentate gyrus were found to express miR-451a. Cells with remarkably intense staining were observed in particular in the subgranular zone and the hilus (**Fig 6A´–6C´**). Quantitative analysis of miR-451a expression in hippocampus revealed an increase from postnatal day 5 through day 15 (**Fig 6E**), although the difference was statistically not significant.

### Genetic ablation of miR-451a influences in vivo neurogenesis at certain postnatal developmental stages

**Fig 7:**
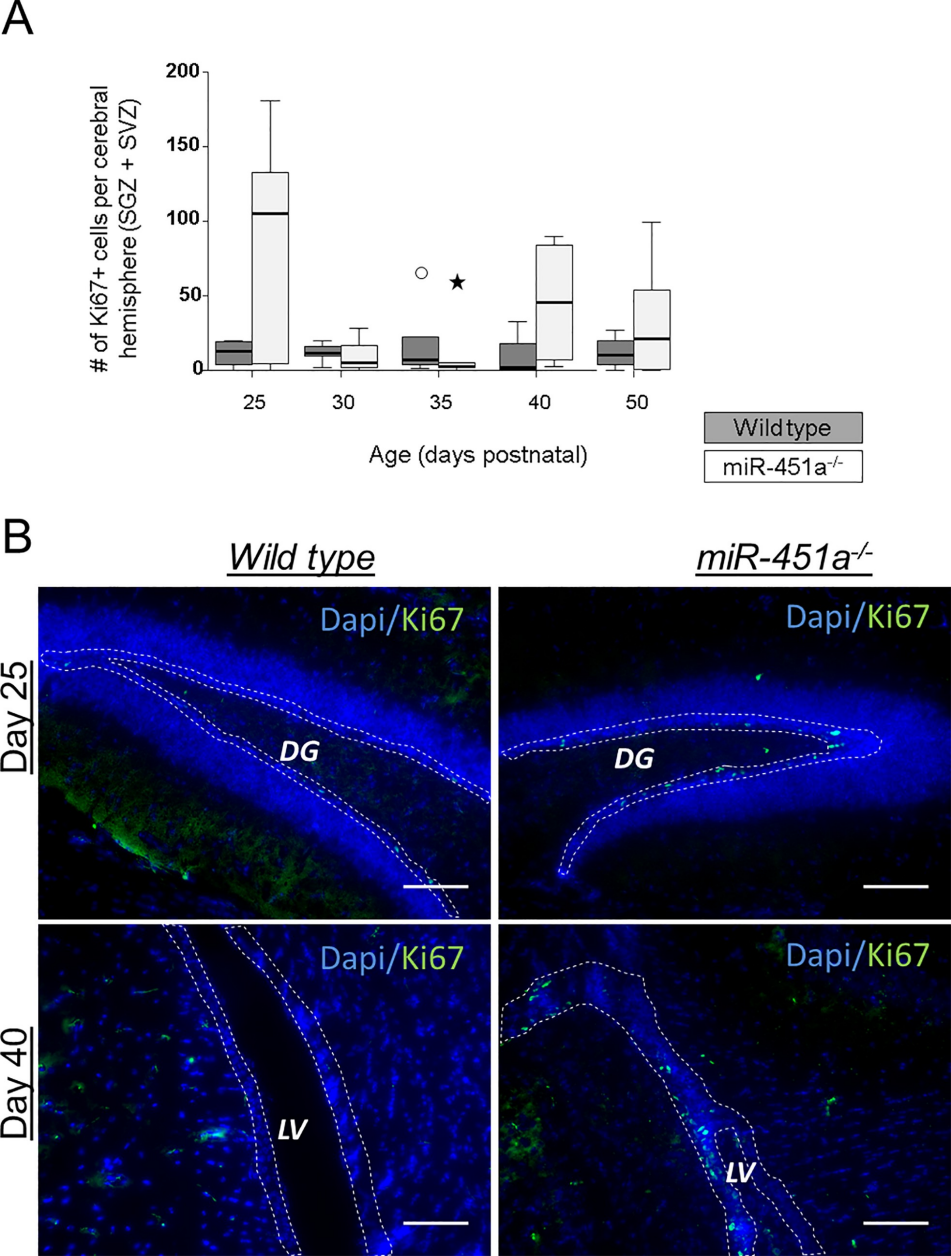
MiR-451a^-/-^ mice show higher cell proliferation in adult neurogenic regions. Proliferating cells were quantified in coronal brain sections of wild type and miR-451a^-/-^ mice at postnatal days 25, 30, 35, 40 and 50 by Ki67 immunostaining (**A**). Markedly higher numbers of Ki67^+^ cells were observed in miR-451a^-/-^ animals, particularly at days 25 and 40 in the subgranular zone (SGZ, demarcated with dashed lines) of the hippocampal dentate gyrus (DG) and the subventricular zone (SVZ, demarcated with dashed lines) of lateral ventricles (LV). Representative microscopy images for day 25 and day 40 animals are shown in (**B**). Cell nuclei were counterstained with DAPI. Immunostaining and quantification were conducted on at least two coronal sections per animal with *n≥4* animals per group per time point. The statistical significance of the observed differences was assessed with the Mann-Whitney U Test. The differences were statistically not significant. Circle: outliers, Star: extreme outliers. Scale bars: 50 μm.

To further verify the role of miR-451a in promoting differentiation, we analysed the expression of proliferation and early differentiation markers (Ki67 and doublecortin (DCX) respectively) in young wild type and miR-451a^-/-^ mice. At postnatal days 25 and 40, marked differences in the number of Ki67^+^ cells were observed between the wild type and miR-451a^-/-^ mice (**Fig 7**), although statistically not significant due to high inter-animal variances. At days 30, 35 and 50 the numbers of Ki67^+^ cells were comparable between wild type and miR-451a^-/-^ animals.

**Fig 8:**
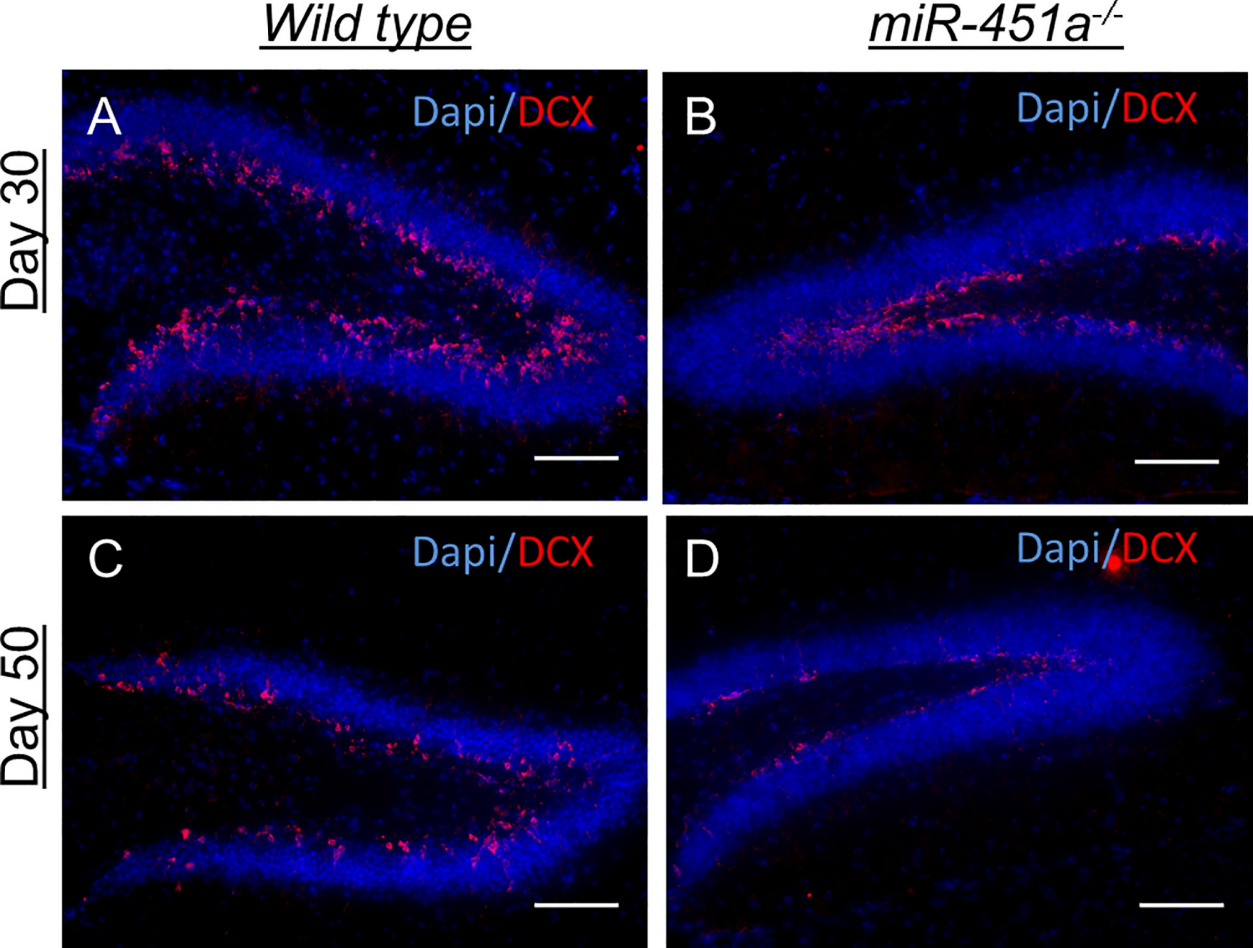
Imbalanced expression of neuronal differentiation marker doublecortin in miR-451a^-/-^ mice. Neuronal differentiation in the subgranular zone (SGZ) of dentate gyrus (DG) in hippocampus was evaluated by doublecortin (DCX) immunostaining of coronal brain sections of wild type and miR-451a^-/-^ animals. Qualitative evaluation revealed markedly lower expression of DCX in the miR-451a^-/-^ group at postnatal days 30 (**A, B**) and 50 (**C, D**). Immunostainings were conducted with at least two coronal sections per animal with *n≥4* animals per group per time point. Scale bars: 50 μm.

DCX expression in the SGZ of hippocampal DG, on the other hand, was lower in miR-451a^-/-^ mice at days 30 and 50 compared to wild type animals (**Fig 8A–8D**). At postnatal days 25, 35 and 40, DCX expression was comparable in both groups (**data not shown**). Taken together, these data suggest that, whilst genetically modified animals tend to have more cell proliferation in the adult neurogenic regions at some time points, fewer of these cells appear to be successfully directed towards a neurogenic fate during the following days. This finding is consistent with the results of our *in vitro* overexpression experiments.

## Discussion

### MiR-451a as modulatory factor in shaping the neuronal phenotype

We detected a time-dependent upregulation of endogenous miR-451a expression during RA-induced neuronal differentiation of NT2/D1 cells, with highest expression at day 28 in neuron-like cells, indicative of a role in neuronal maturation. This observation was in line with a similar miR-451a upregulation during erythroid maturation [71] and impaired erythrocyte maturation upon loss of miR-451a function [15, 54, 72–74]. These findings suggest that miR-451a drives maturation of multiple cell lineages. Our observations with miR-451a overexpressing cells further substantiated this involvement, where mRNA expressions of differentiation markers (GFAP, βIII Tubulin, NF200, DCX and MAP2) [29–32, 75] were upregulated at earlier time points than controls, and the early stem or progenitor marker Nestin [22, 23] was downregulated at days 22 and 28, which contrasted with rather stable expression by control cells at these time points. These molecular changes were, moreover, consistent with morphological observations of miR-451a overexpressing cells, which exhibited longer neurites, and denser and more intricate neurite networks at earlier time points than controls. On the contrary, cells with miR-451a knockdown exhibited opposite effect with neurites shorter in length and less in number, and impaired network formation at day 22 compared to both G-0 controls and miR-451a overexpressing cells. Interestingly, upregulated expression of Nestin, βIII Tubulin and NF200 already at the undifferentiated stage (day 0) in miR-451a overexpressing cells was noteworthy as it indicated initially a higher potential for neural differentiation in these cells, which was reversed in the cells with miR-451a knockdown with downregulation of all three markers at day 0. On the other hand, expression of these three markers was not significantly different than the respective control group at day 22, both groups being similar to AB.G.ct controls. The discrepancy between the morphological observations and marker expression should be based on the masking effect in RNA sampling. Even less in numbers, morphological changes in the heterogeneous (differentiated or undifferentiated) cell mixture in a neurosphere are readily observable when less number of cells is of neuronal phenotype in miR-451a knockdown compared to G-0. But the differences in gene expression might have been masked by the considerable proportion of undifferentiated cells in both groups, therefore not detectable within an RNA pool of heterogeneous cell population. This also explains the still high Nestin expression at day 28 in AB.G.ct cells (~2-fold>AB.G.miR-451a), although they maturity comparable to miR-451a overexpressing group in morphological observations.

MiR-451a overexpression in Glioblastoma multiforme (GBM) resulted in decreased cell proliferation and viability, consistent with a tumour suppressor role [1, 4]. GBM, notably, contains self-renewing, tumorigenic cancer stem cells (CSCs) that contribute to tumour initiation and therapeutic resistance [76–78]. These CSCs could originate from neural stem cells [79] and express neural precursor markers, and are capable of differentiating into tumour cells expressing more mature neural precursor markers [80]. Our results shed light on possible mechanisms of miR-451a tumour suppressor activity in these cells, namely through induction of differentiation and associated reduced tumourigenicity. A decisive role of miR-451a in self-renewal, tumourigenicity and chemoresistance has, indeed, been shown in colonospheres [3], although not in GBM. Assuming similar mechanisms of action of miR-451a in CSCs and stem cells in general, one can legitimately speculate that miR-451a acts as a differentiation factor in these cell types as observed by us during *in vitro* neuronal development.

### Role of miR-451a targets

Six of the ten selected validated miR-451a target genes (MIF, AKT1, CAB39, YWHAZ, RAB14 and TSC1) were consistently downregulated throughout the time course of RA-induced neuronal differentiation of miR-451a overexpressing cells. We checked five of these (MIF, AKT1, CAB39, YWHAZ and TSC1) in cells with miR-451a knockdown and they were upregulated. Of these target genes, MIF, YWHAZ, TSC1 and AKT1 have been shown to be involved in neurogenesis [38, 52, 63, 65]. Notably, MIF has been shown to promote NSC proliferation and to be repressed in NeuN^+^ mature neurons [38] and TSC1 has been reported to exert inhibitory effects on neuronal differentiation [63]. MiR-451a-mediated suppression of both seems to contribute, at least partially, to earlier neuronal maturation in miR-451a overexpressing cells and vice versa its upregulation should partially contribute to delayed maturation upon miR-451a knockdown. On the other hand, YWHAZ and AKT1 are known to promote neuronal differentiation [52, 65]. Although both were expressed significantly lower levels in miR-451a overexpressing cells and higher in miR-451a knockdown group compared to controls, this miR-451a-mediated modulation was not strong enough to prevent/promote neuronal maturation. CDKN2D, another miR-451a target that is necessary for maintenance of neuronal maturity [50], similarly exhibited increased expression at later time points of differentiation, although lower than in control cells. Taken together, these findings suggest that miR-451a-induced neuronal maturation might not be solely mediated by downregulation of its targets. Indeed, downregulation of other predicted (OSR1 and TNS4) or validated targets (PSMB8, CXCL16, CDKN2D and IL6R) at rather late time points (days 22 and 28) of neuronal differentiation strongly suggest that miR-451a-mediated neuronal maturation operated as part of a broader mechanism, but certainly in a context-dependent manner. This presumption has also been underlined by a previous study in which hydroxymethylbilane synthase b (hmbsb), a miR-451a target validated by reporter assays, was not necessarily a physiological target during zebrafish primitive erythropoiesis [74]. MiR-451a-mediated neuronal maturation, therefore, does not seem to depend on a simplistic mechanism comprising modulation of a few target genes, but is rather achieved as a result of an initial change in the molecular make-up reflected by changes in mRNA expression of genes responsible for many different processes, all acting together as a supportive “team” to promote neuronal maturation.

### MiR-451a in neurogenesis in vivo

The molecular role of miR-451a seems to be more of a modulatory one, since we did not observe any obvious anatomical or structural changes in the brains of miR-451a knockout mice. Behavioural tests also did not reveal obvious mental deficits, although miR-451a has been previously correlated with explorative behaviour, learning and memory function [81]. We observed an imbalance between proliferation and maturation in adult neurogenesis in miR-451a knockout mice. A higher rate of cell proliferation was present at some postnatal time points in adult neurogenic regions (SGZ, SVZ) of miR-451 knockout brains, which is in line with previously reported inhibitory role of miR-451a in cell proliferation [82]. However, this was followed by lower level DCX expression during the next 5–10 days, indicating differentiation of the newly generated cells, and maybe their migration as well, should be somehow disturbed. This might in turn create an inhibitory effect on further proliferation until progeny cells leave the proliferating zone (via differentiation, migration, or dying). Indeed, multiple mechanisms have been suggested for the suppression of NSC proliferation via direct feedback mechanisms by the progeny cells, possibly mediated by Notch signaling or neurotransmitters (GABA) (reviewed in [83]). The impaired/delayed differentiation of these cells might extend the inhibitory period, which would explain the resting phases (days 30, 35) in between proliferative phases (days 25, 40, 50) observed in knockout animals. These observations further point to a modulatory role for miR-451a in neuronal maturation *in vivo*. Genetic ablation of miR-451a might, moreover, result in a deployment of compensatory mechanisms. Notably, suppression of neurogenesis by genetic ablation of cyclin D2 in mice did not cause any learning deficits [84], whilst irradiation-induced diminishment of NSCs led to impairment of spatial learning when monitored in an unbiased, automated home cage environment [85, 86], indicative of a strong contrast between genetic ablation and abrupt changes. This might also explain why miR-451a knockout mice did not exhibit neurological deficiency under physiological conditions.

## Supporting information

S1 Fig Ntera2/D1 cells were transduced with a lentiviral vector carrying miR-451a (AB.G.miR-451a) or a control vector (AB.G.ct). Expression of the reporter gene eGFP was indicative of successful transduction. Both groups of cells were sorted according to the magnitude of eGFP fluorescence into high, middle and low eGFP fluorescent cells using fluorescent activated cell sorting (FACS) (**A**). The normalized copy number/20ng miRNA of miR-451a was significantly higher in undifferentiated NT2 cells transduced with AB.G.miR-451a than in cells transduced with the control vector (AB.G.ct) (**B**). Experiments were conducted with 3 biological replicates (*n = 3*). The statistical significance of the differences was assessed with the Mann-Whitney U-test. ***p < 0.001. Error bars represent the standard error of the mean (SEM).
(TIF)

S2 Fig Differential expression of neural precursor and neuronal differentiation markers upon miR-451a knockdown. At undifferentiated stage (day 0) G-U6-451PT transduced cells exhibited lower expression of Nestin (**A**), βIII Tubulin (**B**) and NF200 (**C**) compared to the control group (G-0). At day 22 all three markers showed increased expression in both groups. *n = 3* biological replicates. Statistical significance of differences was tested with Mann Whitney U Test. Error bars show standard error of the mean (SEM).
(TIF)

S3 Fig Neurite formation at day 28 of differentiation. At day 28 of RA-induced differentiation AB.G.ct cells (**A**) exhibited longer neurites than at day 22. The differences were, however, sustained as AB.G.miR-451a transduced Ntera2/D1 cells (**B**) exhibited more intricate and denser neurite networks. Neurospheres were immunostained for Neurofilament heavy chain (NF200). Pictures are representative of at least three different stainings. Scale bars: 100 μm.
(TIF)

S4 Fig mRNA expression of miR-451a targets in G-U6-451PT transduced NT2 cells. mRNA expression of validated target genes of miR-451a were upregulated in cells with miR-451a knockdown at day 0 and day 22 of differentiation. Data is represented as mean fold change compared to control group (G-0). Statistical significance of the changes were tested with Mann Whitney U Test. *n = 3* biological replicates. Error bars show standard error of the mean (SEM).
(TIF)

S1 Table List of primers used for qRT-PCR quantification and their sequences. (DOCX)
