# Supplementary figures and images for: MicroRNA-451a overexpression induces accelerated neuronal differentiation of Ntera2/D1 cells and ablation affects neurogenesis in microRNA-451a-/- mice
Source: PLoS One. 2018 Nov 21;13(11):e0207575. doi: 10.1371/journal.pone.0207575 (PMC6248975; doi:10.1371/journal.pone.0207575)

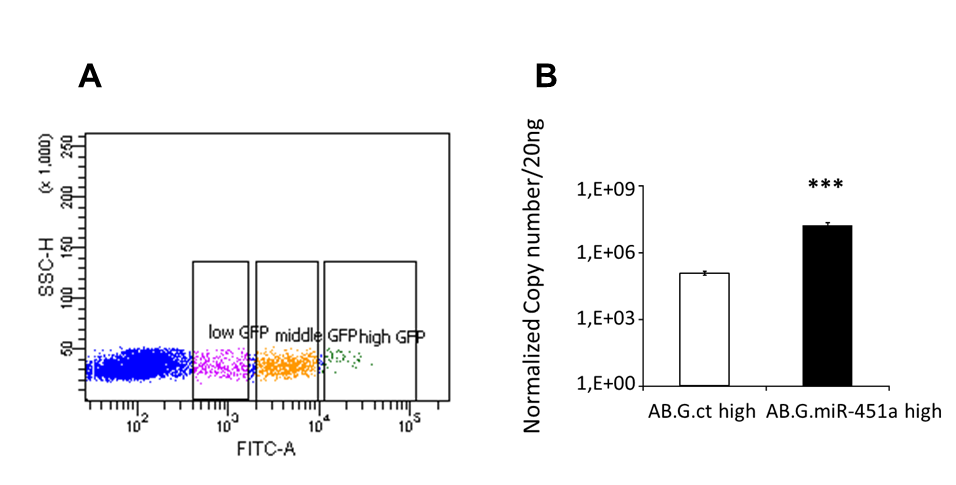

Supplement: S1 Fig — Expression of the reporter gene eGFP was indicative of successful transduction. Both groups of cells were sorted according to the magnitude of eGFP fluorescence into high, middle and low eGFP fluorescent cells using fluorescent activated cell sorting (FACS) (A). The normalized copy number/20ng miRNA of miR-451a was significantly higher in undifferentiated NT2 cells transduced with AB.G.miR-451a than in cells transduced with the control vector (AB.G.ct) (B). Experiments were conducted with 3 biological replicates (n = 3). The statistical significance of the differences was assessed with the Mann-Whitney U-test. ***p < 0.001. Error bars represent the standard error of the mean (SEM). (TIF) [file pone.0207575.s001.tif]

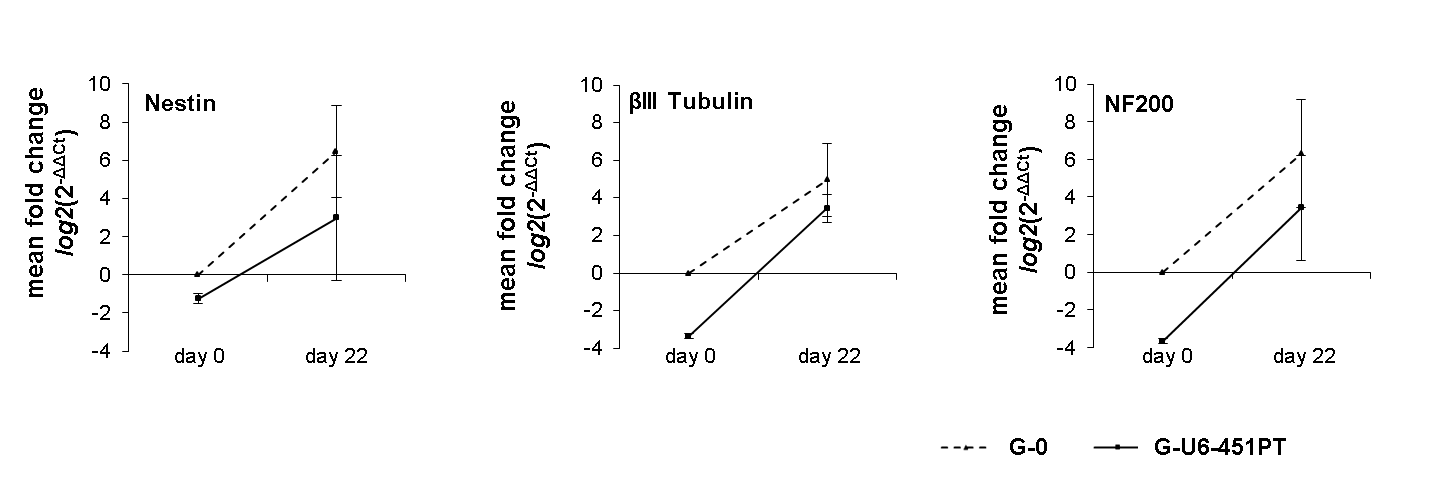

Supplement: S2 Fig — At undifferentiated stage (day 0) G-U6-451PT transduced cells exhibited lower expression of Nestin (A), βIII Tubulin (B) and NF200 (C) compared to the control group (G-0). At day 22 all three markers showed increased expression in both groups. n = 3 biological replicates. Statistical significance of differences was tested with Mann Whitney U Test. Error bars show standard error of the mean (SEM). (TIF) [file pone.0207575.s002.tif]

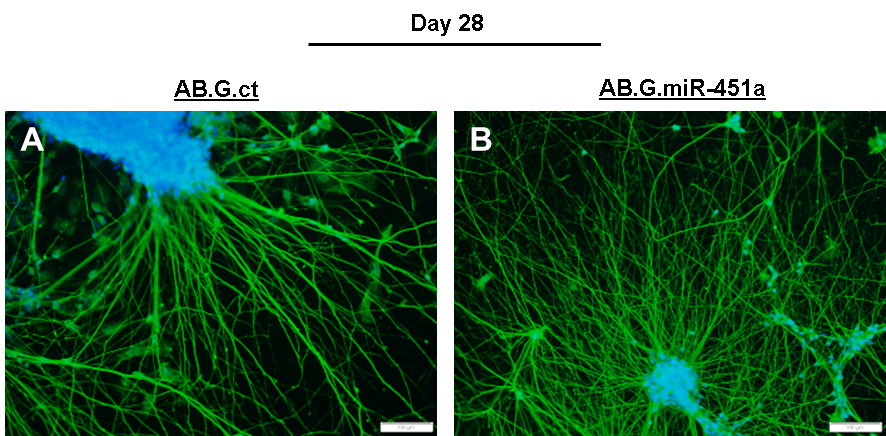

Supplement: S3 Fig — At day 28 of RA-induced differentiation AB.G.ct cells (A) exhibited longer neurites than at day 22. The differences were, however, sustained as AB.G.miR-451a transduced Ntera2/D1 cells (B) exhibited more intricate and denser neurite networks. Neurospheres were immunostained for Neurofilament heavy chain (NF200). Pictures are representative of at least three different stainings. Scale bars: 100 μm. (TIF) [file pone.0207575.s003.tif]

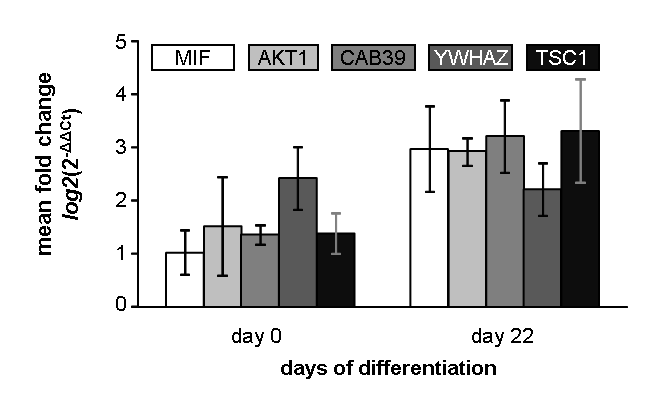

Supplement: S4 Fig — mRNA expression of validated target genes of miR-451a were upregulated in cells with miR-451a knockdown at day 0 and day 22 of differentiation. Data is represented as mean fold change compared to control group (G-0). Statistical significance of the changes were tested with Mann Whitney U Test. n = 3 biological replicates. Error bars show standard error of the mean (SEM). (TIF) [file pone.0207575.s004.tif]
