# Supplementary material for: MicroRNA-451a overexpression induces accelerated neuronal differentiation of Ntera2/D1 cells and ablation affects neurogenesis in microRNA-451a-/- mice
Source: PLoS One. 2018 Nov 21;13(11):e0207575. doi: 10.1371/journal.pone.0207575 (PMC6248975; doi:10.1371/journal.pone.0207575)
Supplement: S1 Table — (DOCX) [file pone.0207575.s005.docx]

S1 Table: List of primers used for qRT-PCR quantification and their sequences.

| **Primer** | **Sequence 5'- 3'** | **Product Size** | **Source** |
| --- | --- | --- | --- |
| Nestin | fw: TCAAGATGTCCCTCAGCCTGGA  rev: AAGCTGAGGGAAGTCTTGGAGC | 106 bp | Origene |
| NF200 (heavy chain) | fw: CTGAGGAACACCAAGTGGGAGA  rev: TCCGACACTCTTCACCTTCCAG | 127 bp | Origene |
| MAP2 | fw: TCAGAGGCAATGACCTTACC  rev: GTGGTAGGCTCTTGGTCTTT | 319 bp | (1) |
| DCX | fw: TATGCGCCGAAGCAAGTCTCCA  rev: CATCCAAGGACAGAGGCAGGTA | 60 bp | Origene |
| βIII Tubulin | fw: GGCAACCAGATCGGGGCCAAGT  rev: CCCTGCAGGCAGTCGCAGTTT | 359 bp | Primer3  Output |
| GFAP | fw: GCCAAGCACGAAGCCAACGAC  rev:GAGCCGGCGGCGTTCCATTTACAAT | 457 bp | Primer3  Output |
| AKT1 | fw: TGGACTACCTGCACTCGGAGAA  rev: GTGCCGCAAAAGGTCTTCATGG | 154 bp | Origene |
| CAB39 | fw: GAGCATGGCTGTTCTGGAAAAGC  rev: GCTACTGCTTCTGTCTGAGGCT | 141 bp | Origene |
| CXCL16 | fw: CCTATGTGCTGTGCAAGAGGAG  rev: CTGGGCAACATAGAGTCCGTCT | 143 bp | Origene |
| CDKN2D | fw: GTGCATCCCGACGCCCTCAAC  rev: TGGCACCTTGCTTCAGCAGCTC | 100 bp | Origene |
| IL6R | fw: GACTGTGCACTTGCTGGTGGAT  rev: ACTTCCTCACCAAGAGCACAGC | 146 bp | Origene |
| MIF | fw: AGAACCGCTCCTACAGCAAGCT  rev: GGAGTTGTTCCAGCCCACATTG | 122 bp | Origene |
| OSR1 | fw: CCTACACCTGTGACATCTGCCA  rev: GTGAGTGTAGCGTCTTGTGGAC | 159 bp | Origene |
| POU3F2 | fw: AAAGGCCAGTTCCCATACCT  rev:TCATTTCCCCCAATGAATGT | 93 bp | Primer3  Output |
| PSMB8 | fw: CCTTACCTGCTTGGCACCATGT  rev: TTGGAGGCTGCCGACACTGAAA | 128 bp | Origene |
| RAB14 | fw: GCGATTTAGGGCTGTTACACGG  rev: CCTTGCATCTGTCAACCAGCTG | 118 bp | Origene |
| TSC1 | fw: CTGGACAGACTGATACAGCAGG  rev: TGCGGATCTCATCTGAAGGAGG | 124 bp | Origene |
| TNS4 | fw: GGACAGCAATGACCTCATCCGA  rev: AATGCTGGCACACGAAGGCAGA | 119 bp | Origene |
| YHWAZ | fw: ACCGTTACTTGGCTGAGGTTGC  rev: CCCAGTCTGATAGGATGTGTTGG | 130 bp | Origene |

1. Yan WH, Cao MD, Liu JR, Xu Y, Han XF, Xing Y, et al. Effects of EGF and bFGF on expression of microtubule-associated protein tau and MAP-2 mRNA in human umbilical cord mononuclear cells. Cell biology international. 2005;29(2):153-7.
